# Challenges and opportunities to improve autism services in low-income countries: lessons from a situational analysis in Ethiopia

**DOI:** 10.1017/gmh.2016.17

**Published:** 2016-07-01

**Authors:** B. Tekola, Y. Baheretibeb, I. Roth, D. Tilahun, A. Fekadu, C. Hanlon, R. A. Hoekstra

**Affiliations:** 1 Department of Life, Health and Chemical Sciences, The Open University, UK; 2 Department of Psychiatry, School of Medicine, College of Health Sciences, Addis Ababa University, Ethiopia; 3 Centre for Global Mental Health, Institute of Psychiatry, Psychology & Neuroscience (IoPPN), King's College London, UK; 4 Department of Psychology, IoPPN, King's College London, UK

**Keywords:** Africa, autism, child, mental health services, stigma

## Abstract

**Background.:**

Little has been reported about service provision for children with autism in low-income countries. This study explored the current service provision for children with autism and their families in Ethiopia, the existing challenges and urgent needs, and stakeholders’ views on the best approaches to further develop services.

**Methods.:**

A situational analysis was conducted based on (i) qualitative interviews with existing service providers; (ii) consultation with a wider group of stakeholders through two stakeholder workshops; and (iii) information available in the public domain. Findings were triangulated where possible.

**Results.:**

Existing diagnostic and educational services for children with autism are scarce and largely confined to Ethiopia's capital city, with little provision in rural areas. Families of children with autism experience practical and psychosocial challenges, including severe stigma. Informants further raised the lack of culturally and contextually appropriate autism instruments as an important problem to be addressed. The study informants and local stakeholders provided several approaches for future service provision expansion, including service decentralisation, mental health training and awareness raising initiatives.

**Conclusions.:**

Services for children with autism in Ethiopia are extremely limited; appropriate care for these children is further impeded by stigma and lack of awareness. Ethiopia's plans to scale up mental healthcare integrated into primary care provide an opportunity to expand services for children with autism and other developmental disorders. These plans, together with the additional strategies outlined in this paper can help to address the current service provision gaps and may also inform service enhancement approaches in other low-income countries.

## Background

Little is known about autism in Africa: the vast majority of autism research studies to date have been conducted in Western, high-income countries (Durkin *et al.* 2015), resulting in a research gap concerning studies from low-income countries like Ethiopia. Due to a lack of epidemiological studies the prevalence of autism in Africa is unknown (Elsabbagh *et al.* 2012). The few autism studies conducted in Africa indicate a lack of knowledge and awareness about autism, inadequate mental healthcare facilities and a severe shortage of trained personnel (Bakare & Munir, 2011). No African country has published policies or good practice guidelines for autism assessment, treatment, education and support (De Vries, 2016). A recent report of an autism meeting attended by 47 delegates from 14 African countries highlighted the lack of available autism services throughout Africa and the need to raise awareness and develop autism screening, training and service strategies on the continent (Ruparelia *et al.* 2016).

Similar to other African countries, Ethiopia has limited autism service provision. The detection of, and care for, children with autism in Ethiopia is further impeded by stigma surrounding mental health (Shibre *et al.* 2006) and misconceptions about the causes of developmental disability and mental illness (Alem *et al.* 1999; Abera *et al.* 2015). We recently examined the experienced stigma, explanatory models and unmet needs of 102 help-seeking caregivers of children with autism and/or intellectual disability (ID) in Ethiopia (Tilahun *et al.* 2016). Caregivers provided a mixture of biomedical (e.g. head injury or birth complications) and supernatural (e.g. spirit possession or sinful act) explanations for their child's condition. Caregivers also reported high levels of stigma, with higher stigma associated with seeking help from traditional institutions, providing supernatural explanations and affiliation to Orthodox Christian faith. The majority (75%) of caregivers reported unmet needs regarding their child's educational provision and many (47%) also indicated an unmet need for support from health professionals. These findings illustrate the great challenges experienced by families with children with developmental disorders in Ethiopia.

In recent years however, Ethiopia's mental healthcare system has become the focus of new initiatives. The National Mental Health Strategy (2012/13–2015/16) presents a plan for scaling up mental healthcare and recognises children with mental disorders as a vulnerable group. Training of mental health specialists is being expanded, with in-country psychiatrist, Ph.D., Masters and psychiatric nurse training programmes, and basic mental health training for rural community-based health workers. New initiatives from local non-governmental organisations (NGOs) also contribute to an increase in autism awareness and service provision in Ethiopia.

Although these developments are promising, existing services for children with autism have scarcely been documented. Moreover, little has been done to explore opportunities and challenges to expand services and the most effective ways for future service development. This paper aims to assess the current health and education service provision for children with autism in Ethiopia. It explores the unmet needs, future opportunities and stakeholders’ views of the best approach to further develop services. Matched with the views from Ethiopian caregivers of children with developmental disorders (Tilahun *et al.* 2016), this paper will serve as baseline work for future studies and service interventions and hopes to also inform capacity building strategies in other low-income countries.

## Methods

### Research design

This study is part of the Health Education and Training+ (HEAT+) project, Ethiopia's first autism research project. A situational analysis was conducted based on (i) qualitative interviews with existing service providers; (ii) consultation with a wider group of stakeholders through two stakeholder workshops; and (iii) information available in the public domain. Findings were triangulated (Thurmond, 2001) where possible, using data triangulation (by interviewing a range of stakeholders), investigator triangulation (the first and third author read the qualitative interview transcripts and independently identified themes) and methodological triangulation (data was used from qualitative interviews, consultation with stakeholders and publicly available information). HEAT+ received approval from the Open University's Human Research Ethics Committee and the Institutional Review Board of the College of Health Sciences, Addis Ababa University.

### Qualitative interviews

Qualitative interviews were conducted with informants (*N* = 10) from existing education and healthcare service providers for autism, including two psychiatrists, a paediatric neurologist, founders and teachers of two autism centres, the deputy head teacher of a school with an inclusive education programme and representatives from two NGOs working with individuals with disabilities in Ethiopia (see Table 1). Participants were recruited through purposive and snowball sampling. Key participants from government-run hospitals and from autism centres were approached first. Further participants were invited following information supplied by the initial participants.

**Table 1.** Overview of participants in qualitative interviews

Service providers	No of participants	Occupation of participants	Years working with children with autism and/or other developmental disorders	Informants referenced in text as
Government hospital	2*	Child and adolescent psychiatrist at Yekatit 12 hospital's child mental health clinic General psychiatrist at Yekatit 12 hospital's child mental health clinic	10 years	*Clinic1/2/3*
			2 years	
Private clinic	1	Paediatric neurologist at a private clinic	30 years	*Clinic1/2/3*
Autism centres	4	Founder of the Joy Center and mother of a child with autism Head teacher at Joy Center Founder of Nehemiah Autism Center (and mother of a child with autism Programme coordinator of Nehemiah Autism Center and father of a child with autism	11 years	*Centre1a/1b/2a/2b*
			10 years	
			2 years	
			1.5 years	
Government school with an inclusive education programme	1	Senior member of staff at Basilios governmental primary school	10 years	*School*
Community-based rehabilitation organisations	2	CBM Country Director in Ethiopia and Sudan Programme coordinator and acting manager of Cheshire Services Ethiopia	20 years	*CBR1/2*
			10 years	
Total number of participants	10			

Note: *one of these participants is also co-author (Y.B.) of this paper.

Individual in-depth qualitative interviews were conducted guided by an interview schedule (see online Supplementary Material), including questions on existing autism services, experiences of families of children with autism and the best ways to improve services in future. All interviews were conducted in Amharic (the official language of Ethiopia) by the first author, either face to face (*N* = 8) or by telephone (*N* = 2); all were audio tape-recorded and lasted 45–60 min. All participants provided their written informed consent.

Interviews were transcribed verbatim into Amharic and then translated into English by the first author. Transcripts were coded using thematic analysis (Braun & Clarke, 2006). Coding was done manually on a computer: a table was created in Microsoft Word, with data on the left and codes and sub-codes on the right (Hahn, 2008). Before themes/codes were identified, the first and third author (B.T. and I.R.) fully read and re-read interview transcripts, independently noted possible themes/codes and quotes, and then pooled their observations. An initial list of codes and themes was developed and reviewed and then refined after further re-reading.

### Consultation with stakeholders through two workshops

HEAT+ project researchers organised two workshops in Addis Ababa bringing together stakeholders in health education and developmental disorders, including representatives from the Ethiopian Ministry of Health, the Ministry of Education, centres for children with autism and/or ID (some of whom were also parents of a child with autism or ID), experts in mental health, autism and health promotion, tutors for community-based health workers and several NGOs including the World Health Organization. The workshops served to gather stakeholders’ views on the existing services for children with autism and the challenges and opportunities to improve these services. The first workshop took place prior to the qualitative interviews and informed our interview schedule. The findings from these interviews were presented at the second stakeholder meeting; the attendees’ feedback and contextualising comments further informed this paper.

### Public domain information

Information available in the public domain was also consulted, including the Ethiopian National Mental Health Strategy (2012/13–2015/16; Federal Democratic Republic of Ethiopia Ministry of Health, 2012), the Ethiopian Health Sector Transformation Plan (2015/16-2019/20; Federal Democratic Republic of Ethiopia Ministry of Health, 2015), the Ethiopian Ministry of Labour and Social Affairs report on the Status of Persons with Disabilities (Federal Democratic Republic of Ethiopia Ministry of Labour and Social Affairs, 2010) and Ethiopia's third (2005/06-2010/11; Federal Democratic Republic of Ethiopia Ministry of Education, 2005) and fifth (2015/16-2019/20; Federal Democratic Republic of Ethiopia Ministry of Education, 2015) Education Sector Development Programme.

## Results

### Policy and legislative framework for autism

Ethiopia is a signatory party to the United Nations Convention on the Rights of Persons with Disabilities and the Convention on the Rights of the Child. The country does not have a national mental health policy or legislation, but developing such legislation is listed in the National Mental Health Strategy (2012/13–2015/16) as a priority.

### Service landscape

Serving a population of over 96 million, Ethiopia has 60 practicing psychiatrists, of whom two are child psychiatrists. In addition there are 461 psychiatric nurses, 14 psychologists (none trained in child mental health), three clinical social workers, and no occupational therapists (updated from MhGAP-Ethiopia Working Group, 2010).

Primary healthcare in Ethiopia is provided by health centres (1/15000–25000 population) and their satellite health posts (1/3000–5,000 population) connected through a referral system. These are staffed with nurses and health officers with satellite health extension workers providing prevention and promotion services to the community. Child health services (outpatient curative care, vaccination and growth monitoring) are given in 62% of the country's health facilities (Federal Democratic Republic of Ethiopia Ministry of Health, 2015). Mental healthcare is currently not provided at most primary healthcare facilities and only available in regional or zonal hospitals, typically staffed with psychiatric nurses. Mental health specialists (general psychiatrists) are only found at the regional hospital level. Psychiatrists working in these settings have received training in child mental health, but are not child mental health experts. There are only two governmental specialised child mental health clinics, in Addis Ababa's Yekatit 12 and St. Paul's hospitals. In addition there are private clinics with some limited child mental health expertise. Each of these specialised clinics is located in the capital and therefore inaccessible to the majority (85%) of families who live in Ethiopia's rural areas.

Services for children with disabilities and preventive and community-based rehabilitation programmes are primarily given by NGOs and religious charities (Federal Democratic Republic of Ethiopia Ministry of Labour and Social Affairs, 2010). As will be further explained below, their work on autism is limited. A situational analysis from 2005 regarding special needs education identified 15 special schools, primarily run by NGOs and 285 special classes attached to mainstream government schools (Federal Democratic Republic of Ethiopia Ministry of Education, 2005). More recent data on the exact number of schools are unavailable, but Ethiopia's fifth Education Sector Development Programme (Federal Democratic Republic of Ethiopia Ministry of Education, 2015) indicates that currently only 4% of children with special needs are enrolled in primary school education. The programme presents plans to increase this rate to 75% by 2019/20, though acknowledges a financing gap (Federal Democratic Republic of Ethiopia Ministry of Education, 2015). Attendants of our stakeholder meetings indicated that some of the special schools, especially those for children with ID (including the private Bruh centre and a school affiliated to the Ethiopian Evangelical Church Mekane Yesus), educate some children with autism. There are two autism-specific schools: The Joy Center hosts 80 children with 500+ children on a waiting list; the Nehemiah Autism Center enrols 40 children with 250 children on their waiting list. Both centres only serve families from Addis Ababa and its surroundings.

**Fig. 1.**
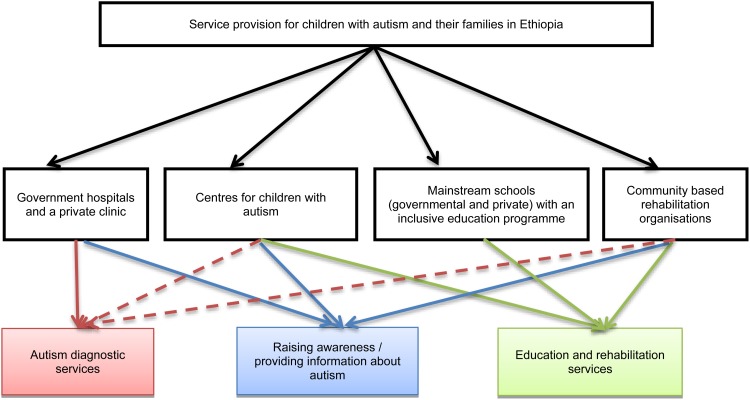
Service providers for children with autism and their families in Ethiopia. Note: Dashed lines refer to informal diagnostic services and diagnostic referral.

In sum, the limited services currently available for children with autism in Ethiopia come from four main types of providers: (i) governmental and private clinics; (ii) centres for children with autism; (iii) mainstream schools with inclusive education programmes; (iv) NGOs and religious charities providing community-based rehabilitation services (see Fig. 1). To further understand the challenges and opportunities related to their activities we conducted in depth interviews with at least one key informant from each type of provider (Table 1).

The analyses of the qualitative interview data generated four core themes: (i) nature and context of the current autism services; (ii) knowledge and understanding about autism; (iii) challenges faced by children with autism and their families; and (iv) identified needs for improvement. Each of these themes is presented below with representative quotes to illustrate findings.

### Nature and context of current autism service provision

The services currently offered by each type of provider will be described in turn.

#### Government hospitals and private clinics

The governmental specialised child mental health clinics and private clinics in Addis Ababa are the only locations where a formal autism diagnosis can be obtained. Government clinics charge a small fee except for people with an exemption certificate (the poorest families in a given administrative unit); private clinics charge a higher fee without exemptions. According to all of our clinical informants, autism diagnoses are based on a clinical interview with the caregiver and a behavioural observation, following diagnostic criteria in the Diagnostic and Statistical Manual of Mental Disorders (DSM-5, American Psychiatric Association, 2013). Standardised diagnostic instruments are rarely used since these have not been adapted and translated for use in Ethiopia, and lack of professional training and resources to pay the license fees prohibit using the English versions even where parents have fluent English.

Families attending the two government child mental health clinics access these services after referral. The majority are poor and live in urban areas; most families living in remote areas have no access to a referral process and even if referred may not be able to travel to Addis Ababa. The families who go to private clinics have a relatively higher income.

Most children receiving an autism diagnosis at the government clinics were reported to be between 4 and 7 years old, more often male, and usually diagnosed with autism co-morbid with ID. Similarly, the private clinic informant indicated rarely seeing cognitively able children with autism. The clinicians reported that parents give a mix of biological (e.g. head injury, perinatal complications, medication during pregnancy, vaccinations or hereditary factors) and spiritual (e.g. punishment from God, curse, or devil's possession) explanations for their child's autism.

### Centres for children with autism

The Joy Center was founded in 2002 by a mother of a child with autism after her child was rejected by many regular schools. Nehemiah Autism Center opened in 2011 after one of the founders experienced similar difficulties finding appropriate schooling for her child.

“…First they promise me to teach him but as soon as they came to know about his condition they told me to take him out. … I used to pay three or four times the usual school fee but no school was willing to keep him”. (Centre1a)

At both centres parents pay a school fee depending on their income, with a limited number of free places available to families who cannot afford the fees. Both centres started with little support, but now receive some financial support from NGOs, businesses and the government. However, three out of four informants from both centres indicated that long term funding is a problem:

“Nobody wants to fund autism. Funders only support illnesses like HIV/AIDS. So lack of sustainable funds is a major challenge”. (Centre 2b)

Most children enrolled are nonverbal, and many have co-morbid conditions such as epilepsy. Both Joy and Nehemiah provide a mix of therapies and training promoting social and communication skills, daily living skills and academic skills. Both centres hold regular parent meetings and present short-term parent training programmes run by local staff and professionals from abroad. Both centres also engage in awareness raising activities including mass walks, fundraising events, conferences, interviews and documentary films. Informants from both centres indicated that these efforts have helped to improve knowledge about autism and to reduce stigma.

“…I appeared in the media with my child to show people that it is OK to have such a child. Then, people started to come. People started to look out for me.” (Center1a)

Recently, the founder of the Joy Center has started training teachers working in ten mainstream schools in the suburbs of Addis Ababa and five schools in Oromia region, to support mainstream schools in providing inclusive education for children with autism.

#### Mainstream schools with inclusive education programmes for children with autism

According to our informant from the governmental primary school Basilios, the school has one special needs classroom hosting 24 children with different developmental disorders including autism. The curriculum focuses on learning the alphabet, numbers, weaving, assembling things and personal hygiene.

Education at mainstream governmental schools is generally free (though registration, uniform and books may incur costs); Basilios school accepts special needs children based on parents’ reports and information from local authorities. When required the school receives medical support from hospitals. The informant indicated the school has a constant turnover of special needs children, for various reasons. Some children drop out because their parents have difficulty bringing them to school; when children are beyond the school's capacity they are sent to other special needs schools, e.g. to a school for deaf children.

#### Community-based rehabilitation organisations

We identified two organisations engaged in community-based rehabilitation activities related to autism in Ethiopia: CBM (previously Christian Blind Mission) works to improve the quality of life of people with disabilities and has been active in Ethiopia for over 20 years; Cheshire Services Ethiopia, founded in 1962, provides orthopaedic and social rehabilitation services for children with disabilities. Community-based organisations serve as a bridge between mental health experts and the community and also work in rural areas. Following training by psychiatrists and psychiatric nurses, both organisations are involved in identifying and referring children with autism. They also provide community members with training on community-based rehabilitation interventions such as daily living and social skills, so that they can work with local families. However, informants from both organisations conceded that their resources for autism-related work were limited.

“…generally our impact is very small with regards to autism… recently what our people have started to do is when they suspect that the problem is autism they refer the child saying we don't have the capacity to intervene.” (CBR1)

### Knowledge and understanding about autism

The second core theme identified concerns the lack of knowledge and understanding about autism in Ethiopia. While the first clinical autism diagnosis was formally documented in 1986 (Gebre-Mariam, 1986), clinical informants reported that it was not until after the start of the postgraduate psychiatry training at Addis Ababa University in 2003 that autism became more widely recognised among health professionals.

“…when child psychiatrists came here to see the patients they started to introduce us to the diagnosis of autism. So I think it [recognition of autism in Ethiopia] is related to the start of the postgraduate programme in psychiatry.” (Clinic1)

Around the same time the Joy Center was opened. As described above, the awareness raising activities of the autism centres have likely contributed to increased autism awareness. Earlier, children showing symptoms of autism would typically be misdiagnosed with ID. Informants indicated that the majority of children with autism and/or ID still remain without any formal diagnosis, due to lack of appropriately trained staff and lack of awareness, especially in rural Ethiopia. Nevertheless, the key informants concurred that the number of children receiving an autism diagnosis is gradually increasing.

“…because of the increase in awareness and because people hear that there are individuals who work with children with autism they come from different directions: not only from Addis Ababa but from all parts of Ethiopia, even from Somalia.” (Clinic2)

### Challenges faced by children with autism and their families

The third core theme encompassed two subthemes: psychosocial challenges and practical challenges.

#### Psychosocial challenges

Interviews with informants from the autism centres and community-based rehabilitation organisations indicated that children with autism and their families frequently experience exclusion, stigma and negative judgements from others. One informant described how some children were disregarded even by their own parents:

“When we do our outreach programme in 39 towns …many people come to seek help…Some of the children we see have not seen sunshine before….we used to first go door to door and do census before we start our job. When we asked parents whether there are children who have problems in their house, they didn't tell us about their child who has autism or ID. They only showed us their child who has a physical disability. When we said: ‘what about this child?’ they would say this one is useless and *Enkuf* [‘idiotic’]” (CBR2)

One informant from an autism centre described how an ‘educated father’ of four children, when asked how many children he had, answered ‘three’ as ‘he doesn't consider his child with autism as a child’. The same informant indicated that children with autism were sometimes forced to leave public buses, and taxis were not happy to take them. Informants from the autism centres indicated that it is difficult for parents to find rental accommodation because as soon as landlords know about their child's condition, they would force them to leave. The autism centres experienced similar problems:

“When we told them [the property owners] what we were going to do with the house nobody was willing to rent out their house to us. They believed that these children would bring a curse to them and their families so it was very difficult to find a house that we could rent for the school.” (Centre2a)

Parents, especially mothers, are often blamed for their child's autism:

“People tell you this happened because you did not do such and such things for your child; because you didn't take him to the holy water, because you didn't pray for him etc.” (Centre1a)

Two of the key informants are mothers of a child with autism. Both mothers and four out of eight of the other informants indicated that mothers tend to be the main carer, often with little support from others. Attendants of the second stakeholder meeting also raised the challenge of lack of support. When no support is available, some parents resort to chaining up their child at home when they have to go out. It was stressed that parents usually feel they have no alternative but to use chains, to protect their children from harming themselves when there is no supervision.

#### Practical challenges

Due to a lack of provision and limited awareness, getting an autism diagnosis is a challenge. One clinical informant indicated that the relatively low numbers of autism cases they see is due not only to low public awareness but also to ‘weakness’ among health professionals in identifying autism, as ‘autism is a relatively new phenomenon for us’. In addition informants highlighted the problem of accessing appropriate education. Children with autism are often not accepted at mainstream schools or are expelled.

### Identified needs for improvement

Partly informed by our guiding question, ‘identified needs for improvement’ emerged as a core theme in the qualitative interviews. To improve case identification and referral rates, one medical informant suggested providing education and training for community-based health workers. With regards to diagnostic and educational provision, informants put forward two views. Three informants believed that expanding specialist centres is vital, for instance by creating ‘satellite centres’ in rural towns. One clinician put forward that sending children with autism to a mainstream school ‘brings more disadvantages than advantages’ because the lack of professional support results in the child stalling and ‘this brings a sense of failure both to the parent and the child’. In contrast, another doctor believed that an integrated service is more relevant to Ethiopia than specialist autism centres:

“I believe learning is not only vertical but also horizontal. Autistic children can learn a lot from other children. So I believe it is useful to advocate for integrated services. It is also cheaper than establishing a specialist centre.” (Clinic1)

This informant also argued that rehabilitation is best achieved by training caregivers:

“In North America and Europe special schools are needed because nobody spends time at home. But in our country the majority of women are housewives.… If we can give the trainings … to daughters and mothers we can reach many people.” (Clinic1)

This decentralised approach, in which mental health services are available at local hospitals and health centres and basic psychosocial care and intervention is offered by community-based health workers, is also promoted in The Ethiopian National Mental Health Strategy (2012/13–2015/16).

Lastly, medical informants highlighted the need for culturally appropriate screening and diagnostic tools:

“With regards to ID… there is no IQ test in this country. …We can't just bring Western instruments here and apply them without making them socially and culturally relevant… If you show a fork to a child who comes from a rural area he won't recognise it but this doesn't mean … his IQ is subnormal. It is because he has not seen a fork before. … That is why we need an instrument which is relevant to us culturally” (Clinic2)

Based on our informants’ views, the outcomes of stakeholder consultations and initiatives already underway, Table 2 summarises the main needs for improvement in Ethiopia and a way forward.

**Table 2.** Summary of main challenges and potential future directions with regards to service provision for children with autism and their families in Ethiopia

	Main challenges/unmet needs	Potential future directions
1.	Lack of knowledge and understanding about autism among the general public and health professionals	Increase/continue awareness raising activities by autism centres, community-based rehabilitation organisations and other stakeholders.
2.	Lack of culturally and contextually appropriate diagnostic and screening instruments	Translation and validation of the ADOS to Zulu in South Africa (Grinker *et al.* 2012) and to Kiswahili in Kenya (Petrus de Vries, personal communication) currently underway; their experiences can inform future translation and adaptation strategies. An international working group aims to develop open source screening and diagnostic instruments that can be applied globally (Durkin *et al.* 2015). A study to inform the cultural adaptation of an existing autism screening instrument for use in Ethiopia is currently being conducted.
3.	Inadequate mental health services in rural and urban areas	The Ethiopian National Mental Health Strategy (2012/13–2015/16) promotes a decentralised approach in which mental health services are available at local hospitals, district and regional health centres and tertiary facilities.
4.	Lack of funding for autism services; competing public health priorities	The new Sustainable Development Goals (SDGs) explicitly include mental health, generating momentum and opportunity for the scale-up of global mental health (Patel *et al.* 2016), including autism services. The current Ethiopian National Mental Health Strategy already mentions children as a vulnerable group; this recognition together with the momentum for sustainable global mental health development is likely to help in improving services for children with autism in future.
5.	Shortage of healthcare professionals trained in autism at all levels, including psychiatry, clinical psychology, primary healthcare workers and community-based health workers	Training of mental health specialists is being expanded, with an in-country psychiatrist training programme (Alem *et al.* 2010), a Ph.D. programme in mental health epidemiology, Masters programmes in clinical psychology and mental health, and psychiatric nurse training programmes. Rural community-based health workers are upgrading their training studying a 2-week module on mental healthcare including material on autism and other developmental disorders (see also www.open.ac.uk/heat).
6.	Shortage of schools for children with autism/problems of accessing education and intervention for children with autism	Expanding specialist centres for children with autism e.g. creating satellite centres in rural areas; expanding integrated services for children with autism in mainstream schools, capitalising on the plans set out in Ethiopia's fifth Education Sector Development Programme (Federal Democratic Republic of Ethiopia Ministry of Education, 2015); training caregivers about autism; training school staff on child mental health and developmental disorders. A draft parent skills training programme developed by WHO for caregivers of children with developmental disorders will be adapted and piloted in Ethiopia.
7.	Stigma, exclusion and negative attitudes/judgments against children with autism and their families	The 2 week module studied by rural community-based health workers (see under 5.) includes guidance on conducting awareness-raising and anti-stigma campaigns; continue awareness raising work by autism centres, community-based rehabilitation organisations and other stakeholders.
8.	Lack of research studies of autism in Ethiopia	The Ph.D. programme in mental health epidemiology at Addis Ababa University is training a future cohort of Ethiopian mental health research experts. Through the HEAT+ project our research team is conducting the first autism research studies in Ethiopia.

## Discussion

The situational analysis presented in this study provides insight into the current services for children with autism in Ethiopia. Our results indicate there is lack of knowledge about autism in Ethiopia. The diagnostic and education provision is extremely limited. Most services are confined to the capital and have long waiting lists. Moreover, families face severe psychosocial and practical challenges in caring for their child with autism, including stigma and social exclusion.

Many of these problems are not unique to Ethiopia. Little is known about the aetiology and characteristics of autism in other African countries (Bakare & Munir, 2011) and inadequate autism services and trained personnel are a widely reported problem throughout the continent (Ruparelia *et al.* 2016). Funding is a major barrier to improving mental health services (Saraceno *et al.* 2007) and special needs education provision (Federal Democratic Republic of Ethiopia Ministry of Education, 2015). Our informants from the two autism centres indicated funding to be a major challenge. The other informants did not actively raise financial constraints – most likely because they assumed this problem to be obvious. As indicated by our autism centre informants, competing priorities may contribute to the lack of funding. Other public health priorities such as HIV/AIDS, malaria and tuberculosis mean that governments and international donors give little attention and funding to mental healthcare (Breuer *et al.* 2016). Generally low-income countries allocate less than 1% of their overall health budget to mental healthcare (World Health Organization, 2014).

The importance of culturally and contextually relevant diagnostic tools has been highlighted before (Zhang *et al.* 2006; Daley *et al*. 2012; De Vries, 2016), and the challenges and opportunities in developing such tools for low-resource settings were described by Durkin *et al.* (2015). Recent initiatives to address this problem are highlighted in Table 2.

Our informants’ reports regarding parents’ explanatory models are consistent with findings of our study in Ethiopian caregivers of children with developmental disorders (Tilahun *et al.* 2016), and studies in other sub-Saharan African countries (Bakare & Munir, 2011; Gona *et al.* 2015), showing that parents attribute their child's autism to both biomedical and supernatural/religious factors. The reports that Ethiopian families affected by autism experience exclusion, stigma and negative attitudes complement the findings from our study in caregivers. While Tilahun *et al.* (2016) described stigma experience of caregivers of diagnosed children, our present study shows that severe stigma and exclusion are equally strongly observed in contexts where children have not (yet) been identified and diagnosed.

Our study suggests that mothers usually take the main responsibility of caring for their child. Since mothers are also often blamed for their child's autism and experience negative attitudes from others they are isolated from social life. A Turkish study reported similar findings (Koydemir-Özden & Tosun, 2010). Autism advocacy by parents has contributed to improved public awareness about autism and educational opportunities for children with autism. Many advocacy efforts used the popular media. These initiatives, especially using radio, likely have a wide reach even in remote areas.

It should be acknowledged that the current study focused on the perspectives of informants from health and educational sector services and did not include traditional and religious healers. We recognise that they have an important function in Ethiopian society and may provide additional autism service provision not included in this paper.

## Conclusion

Our study identified four types of autism service providers in Ethiopia: clinics; autism centres; schools with inclusive education programmes; and community-based rehabilitation organisations. Most of these service providers are located in Addis Ababa and inaccessible to the majority of the population living in rural areas. There is a great lack of autism awareness and stigma levels are high. Besides improving service provision there is a need for culturally and contextually appropriate autism instruments. The strategies outlined in this paper (see also Table 2) can help to address these gaps in future and may also inform service enhancement approaches in other low-income countries.

## Supplementary material

For supplementary material accompanying this paper visit http://dx.doi.org/10.1017/gmh.2016.17. click here to view supplementary material
