# Supplementary material for: Challenges and opportunities to improve autism services in low-income countries: lessons from a situational analysis in Ethiopia
Source: Glob Ment Health (Camb). 2016 Jul 1;3:e21. doi: 10.1017/gmh.2016.17 (PMC5454792; doi:10.1017/gmh.2016.17)
Supplement: Supplementary file 1 [file S2054425116000170sup001.docx]

**Supplementary material**

**Qualitative interview schedule**

*For centres/schools/community-based rehabilitation organisations*

*About the centre/institution and the founder’s personal experiences*

- When did you first hear about autism?
- When did you open the centre?
- What motivated you to open the centre (what was the reason/s behind the opening of the centre)?
- Tell me about the process of opening the centre?
- Did you get support from the government and other institutions?
- How many students attended when you first opened the centre?
- How many staff did you have?
- How many students are currently enrolled in the centre?
- How many staff do you have now?
- What types of services do you provide for children with autism and their families?
- How much is the monthly fee?
- Do you now get support from the government and other institutions?
- Do you work with the government and other institutions?
- Your school is based in Addis Ababa, could you tell me about the provision outside Addis Ababa for children with autism and their families?
- What is the centre’s relationship with families of children with autism (probe: do you work with them, do you involve them in decision making, management etc.)?
- What kinds of challenges did/do you face?
- What are your future plans?

*About families of children with autism*

- Tell me about how parents usually came to know (have heard) about your centre?
- What are the experiences of families of children with autism? (probe: the difficulties they face before and after they enrolled their child, coping strategies)
- What are the common explanatory models parents have about autism?
- How do parents treat their child with autism (what is their attitude towards their child with autism)?
- What treatments (not provided by the centre) do parents try for their children with autism?
- Does the attitude of families towards their child with autism change over time? (If yes, how? If No, why?)
- Do parents face stigma because of their child with autism? (If yes, how?)
- What is the attitude of other people towards children with autism and their families?
- Does the attitude of other people towards children with autism and their families change over time? (If yes, how? If No, why?)

*For health professionals*

- What do we know about autism in Ethiopia? (e.g. estimate of prevalence rate; presentation of autism symptoms: to what extent are they similar or different from the main symptoms and comorbidities described in developed countries)
- What is the process for diagnosing a child with autism in Ethiopia?
- What do you do when a child is diagnosed with autism? (What kind of support/service is available?)
- What is the typical reaction of parents when their child is diagnosed with autism?
- What types of services do children with autism and their families often use? (Probe: traditional, modern)
- What kind of people come to the clinic to seek support (Probe: level of education, class, age of parents)
- What are the common explanatory models parents have about autism?
- Do parents face stigma because of their child with autism? (If yes, how?)
- What kinds of services are available outside Addis Ababa for children with autism and their families?
- What do you think needs to be done to improve the support for children with autism and their families? (ask for priorities for i) urban areas (Addis Ababa) and ii) rural areas)
